# *Most* quantifiers have *many* meanings

**DOI:** 10.3758/s13423-024-02502-7

**Published:** 2024-05-08

**Authors:** Sonia Ramotowska, Julia Haaf, Leendert Van Maanen, Jakub Szymanik

**Affiliations:** 1 Institute for Logic, Language and Computation, University of Amsterdam, Science Park 107, 1098 XG Amsterdam, The Netherlands; 2 Department of Psychology, University of Potsdam, Karl-Liebknecht-Str. 24/25, 14476 Potsdam, Germany; 3 Department of Experimental Psychology & Helmholtz Institute, Utrecht University, Heidelberglaan 1, 3584 CS Utrecht, The Netherlands; 4 Center for Mind/Brain Sciences Department of Computer Science, University of Trento, Corso Bettini 31, 38068 Rovereto (TN), Italy

**Keywords:** Quantifiers, Vagueness, Response error, Hierarchical Bayesian model

## Abstract

In this paper, we investigate, by means of a computational model, how individuals map quantifiers onto numbers and how they order quantifiers on a mental line. We selected five English quantifiers (*few*, *fewer than half*, *many*, *more than half*, and *most*) which differ in truth conditions and vagueness. We collected binary truth value judgment data in an online quantifier verification experiment. Using a Bayesian three-parameter logistic regression model, we separated three sources of individual differences: truth condition, vagueness, and response error. Clustering on one of the model’s parameter that corresponds to truth conditions revealed four subgroups of participants with different quantifier-to-number mappings and different ranges of the mental line of quantifiers. Our findings suggest multiple sources of individual differences in semantic representations of quantifiers and support a conceptual distinction between different types of imprecision in quantifier meanings. We discuss the consequence of our findings for the main theoretical approaches to quantifiers: the bivalent truth-conditional approach and the fuzzy logic approach. We argue that the former approach neither can explain inter-individual differences nor intra-individual differences in truth conditions of vague quantifiers. The latter approach requires further specification to fully account for individual differences demonstrated in this study.

**Supplementary Information:**

The online version contains supplementary material available at 10.3758/s13423-024-02502-7.

## Introduction

In English, like many other languages, one can express numbers and quantifiers (*many*, *few*, *most*, *some*, and *at least 5*). Researchers have been trying to establish the link between quantifiers and the mental number line (e.g., Hammerton, 1976; Newstead et al., 1987; Pezzelle et al., 2018; Abbondanza et al., 2021). In this study, we developed a computational model to investigate the mapping between numbers and quantifiers. Firstly, we quantified between-individual variability in the quantifier-to-number mapping to establish how flexible this mapping is. Secondly, we tested whether participants put quantifiers on the number line in the same order according to their associate quantity.

### Psycholinguistic studies on quantifiers

Quantifier-to-number mapping has been extensively studied for psychometric purposes (see Moxey and Sanford (1993) for review) to assess whether experimental scales constructed using quantifiers are rank or interval and how distinguishable items of these scales are Moxey and Sanford (1993). For example, Hammerton (1976) found that while between-subject variability in quantifier-to-number mapping is high, individuals tended to rank order quantified sentences consistently. Newstead et al. (1987), in turn, found that participants were less consistent in the usage of low-magnitude quantifiers (e.g., *few*, *several*) than high-magnitude quantifiers (e.g., *many*, *most*). More recently, Pezzelle et al. (2018) investigated the order of quantifiers on a mental line of numbers by measuring the range of proportions covered by each quantifier. They established a consistent order of quantifiers, however, they also showed that the ranges of proportions covered by quantifiers highly overlap, and the high-magnitude quantifiers are less distinguishable. Overall, the psychological studies suggest that despite high individual differences in quantifier-to-number mapping, quantifiers are put in the same order on a scale.

### Semantic approach to quantifiers

Traditionally, formal semantics analyses the meaning of quantifiers in terms of bivalent truth conditions (e.g., Generalized Quantifier Theory, Barwise and Cooper (1981); Mostowski (1957)). The truth condition of a quantifier specifies a **threshold** above or below which the quantifier is true^1^. Some quantifiers like *more than half* have a clear threshold. For example, in the sentence “*More than half* of the As are B” the threshold equals half of set A. Other quantifiers, like *many*, have various thresholds depending on the context Partee (1989). In this study, we use thresholds as a measure of quantifier-to-number mapping.

In addition to thresholds, quantifiers can differ in how precise their meaning boundaries are. We will refer to this phenomenon as **vagueness**. The role of vagueness in natural language has been extensively debated in the linguistic and philosophical literature (e.g., Douven, 2019; Glöckner, 2006; Solt, 2015). Vagueness expresses the intuition that meaning boundaries are gradable. Quantifiers like *many* and *few* do not have a specific threshold. The lack of a specific threshold correlates with borderline cases which constitute a key characteristic of vagueness. For example, if we agree that the sentence “*Many* of the students failed an exam." is true when 20% of students failed, we will also probably agree that the sentence is true when 19% failed. Thus, the threshold for accepting a statement as true for *many* and *few* is fuzzy even given a fixed context (Solt, 2011).

Both threshold and vagueness could give rise to individual differences. Participants might disagree about the threshold for a given quantifier, which results in between-subjects differences in thresholds. Moreover, participants could differ in how consistent they are about the threshold, which results in between-subjects differences in vagueness. While individual differences in categorization of vague concepts have been studied (e.g., Verheyen et al., 2019), they are somewhat neglected in the domain of quantifiers. This could be because vagueness cannot be fully captured by bivalent semantics. Zadeh (1983) proposed to treat quantifiers as fuzzy concepts in which truth conditions take a value in [0, 1]. Therefore, investigation of individual differences in threshold and vagueness of quantifiers is critical for understanding the nature of quantifier representations.

Vagueness and threshold are difficult to separate in experimental studies. For example, according to semantic analysis (see Hackl (2009)), *most* and *more than half* have the same threshold, namely 50%. However, Solt (2011) observed that in certain contexts *most* seems to be inappropriate to use when referring to a proportion slightly above 50%. As one explanation, Kotek et al. (2015) argued that *most* and *more than half* have the same threshold and the observed differences is only due to vagueness (cf., Solt, 2011; Carcassi and Szymanik, 2021). Others Denić and Szymanik (2022); Ramotowska et al. (2023) argued that *most* and *more than half* have different thresholds and in addition, *most* is vague. The observed response differences in the experiment could be attributed to both different thresholds of the quantifiers and differences in vagueness. To test these two effects independently, we propose to measure threshold and vagueness by mapping them onto two different parameters of a computational model.

Additionally, individual differences in task performance hinder the interpretation of behavioral data. Depending on task difficulty and some properties of quantifiers, participants make mistakes which we refer to as **response errors**. For example, participants make more mistakes in truth value evaluation of negative quantifiers (*fewer than half*, *few*) for which the threshold constitutes an upper bound of the scale than positive quantifiers (*more than half*, *many*) for which threshold constitute a lower bound of the scale (Szymanik & Zajenkowski, 2013; Deschamps et al., 2015; Schlotterbeck et al., 2020). Importantly, the higher error rate for negative quantifiers is independent of the proportion against which they were verified (Deschamps et al., 2015). This effect, also known as the polarity effect, was replicated also in other languages than English, for example, in German (e.g., Grodzinsky et al., 2021) and Dutch (e.g., Potthoff et al., 2023). Therefore, we include response error as a third parameter in our model.

The basis for our model is the logistic regression model which is suitable for modeling threshold variability (Verheyen and Égré, 2018; Ramotowska et al., 2020). The three-parameter logistic regression model assumes that the probability that participants verify a statement as true or false depends on the proportion that was presented on a particular trial and the values of the logistic function parameters asymptote, midpoint, and scale:

1 $$\begin{aligned} \text {response} = \frac{\text {asymptote}}{1 + \exp \big ( - \frac{\text {midpoint} - \text {proportion}}{\text {scale}} \big )} \end{aligned}$$

**Fig. 1:**
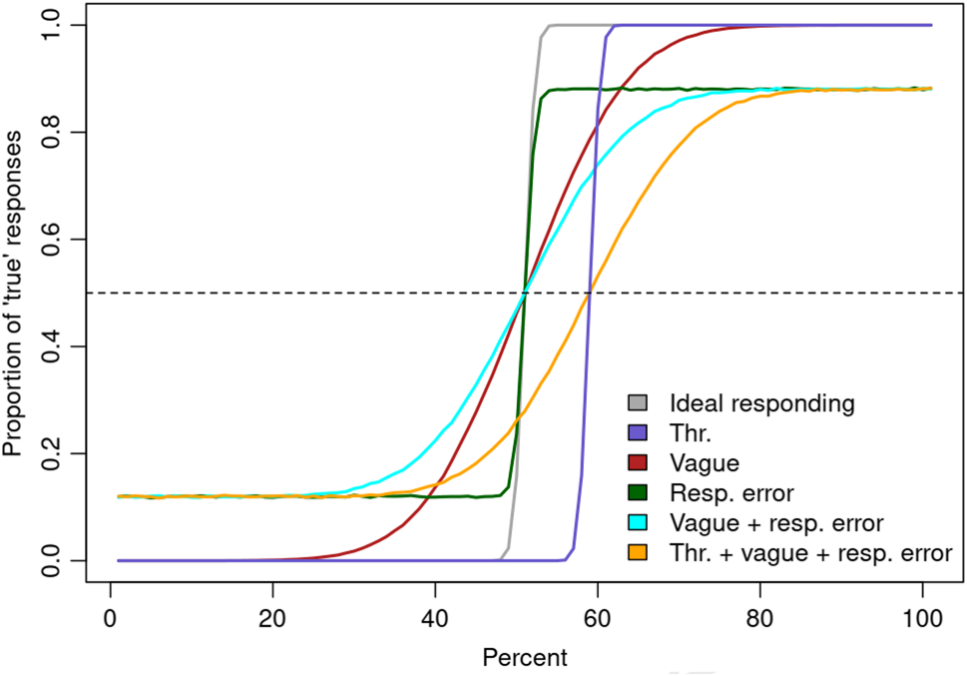
Predicted logistic curves under different threshold (thr.), response error (resp. error), and vagueness (vague) parameters. The *dashed line* indicates the 0.5 proportion of true responses. The percentage for which the logistic curve crosses the dashed line is the threshold

Figure 1 highlights how our parameters threshold, response error, and vagueness map onto the parameters of the logistic regression. For the quantifier *more than half*, for example, ideal responding is achieved when the proportion of ‘true’ responses below 50% is zero, and above 50% is one. The logistic curve has a sharp shape, indicating a rapid shift from false to true responses with a midpoint parameter corresponding to the threshold of 50%. Individual differences in threshold mean that the midpoint (0.5 proportion of true responses) of the logistic curve shifts towards left or right. When the shape of the logistic curve is sharp, participants endorse bivalent truth-conditional semantics. When the responses are affected by vagueness, the perceived threshold varies from trial to trial, and the logistic curve increases gradually corresponding to an increased scale parameter. The response error, in turn, does not change the shape of the response curve. Instead, it lowers the probability of the true response above the threshold and increases the probability of the true response below the threshold equally for all proportions corresponding to the asymptote parameter.

### Current study

We chose five proportional quantifiers, with different degree of potential vagueness, not vague quantifiers (*fewer than half* and *more than half*); vague quantifiers (*few* and *many*); and one quantifier with a debatable status (*most*). Using a cluster analysis on the threshold parameter, we tested if the variability of individual thresholds and the distance between quantifiers on a mental line can be systematically explained by subgroups of participants. Previous studies (e.g., Bott and Noveck, 2004) have shown that participants can form groups with different interpretations of quantifiers. For example, some participants have a literal interpretation of this quantifier (*some and possibly all*, logical responders group) and have an upper-bounded interpretation (*some but not all*, pragmatic responders group). Similarly, *most* could be interpreted as a synonym of *more than half* or as a quantifier indicating a proportion significantly greater than *more than half* (cf. Solt, 2016). Based on the studies discussed above, we hypothesize that the cluster analysis will distinguish at least two subgroups: one with a 50% threshold and one with a higher threshold. We also predicted between-subjects variability in thresholds of *few* and *many*. We expected that participants would choose a smaller proportion for the threshold of *few* than of *many*, however, the vagueness of these quantifiers would lead to disagreement concerning the threshold-to-number mapping and the numerical distance between the thresholds of these quantifiers. In addition, negative quantifiers (*few*, *fewer than half*) can be linguistically analyzed as negations of positive quantifiers (e.g., *fewer than half* means *not more than half*, and *few* means *not many*). The meanings of polar-opposite quantifiers depend on each other Heim et al. (2015). While some participants might treat *few* as a negation of *many*, others might endorse a semantic gap between these quantifiers (Égré & Zehr, 2018) and judge some proportions as neither *few* nor *many*. No such gap is expected for the non-vague pair of polar-opposite quantifiers (*fewer than half* and *more than half*). Because neither semantic theories nor empirical findings predict a specific number of subgroups for *few* and *many*, we applied a data-driven approach to determine the number of clusters. Finally, we predicted that all participants should have a 50% threshold for *fewer than half* and *more than half*. Based on the previous psycholinguistic studies on quantifiers introduced above, we predicted between-subject consistency in the order of quantifiers.

Concerning the vagueness parameter, we predicted that it would reflect the distinction between vague and not vague quantifiers. Égré (2017) argued that the vagueness of a linguistic expression might persist even when there is no uncertainty about the representation of a magnitude. In contrast, the computational model of van Tiel et al. (2021) assumed that the crisp truth-conditional meanings of quantifiers are captured by a threshold parameter, while imprecision in the usage of quantifiers was achieved by incorporating approximate number representations (Dehaene, 1997) into the model. Therefore, the vagueness in their model was a byproduct of uncertainty about perceived magnitude. When a quantifier is evaluated against a magnitude given perceptually, it is difficult to distinguish these two sources of vagueness. In this study, the magnitudes were given as precise percentages, therefore, we expected that the vagueness parameter would reflect an imprecision of quantifier meaning. While both vagueness and response error account for noise in behavioral data, they capture a different aspect of participants’ performance. Response errors as a measure of the quality of task performance (e.g., mistakes, attention lapse) should be participant-specific and, therefore, correlated across quantifiers. In addition, because the verification of negative quantifiers is more error-prone, the response error rate might be higher for *few* and *fewer than half* than other quantifiers. In contrast, vagueness should be quantifier-specific, therefore higher for vague quantifiers and not necessarily correlated across quantifiers. In correlation analysis, we tested whether all parameters of our model make a unique contribution to explaining participants’ behavior.

## Method

### Data availability

The data and analysis code are available at https://github.com/jstbcs/pling-quant. The data analyzed in the paper were previously published by Ramotowska et al. (2023), however, the model reported here was developed independently of that analysis.

### Participants

We recruited 90 English native speakers via the online recruitment platform Amazon Mechanical Turk. We included 71 participants (47 male, age *M* = 35, range 22–59) in the final sample. Subjects gave informed consent before participating in the experiment. The study was approved by the Ethics Committee of the University of Amsterdam’s Faculty of Humanities.

### Experimental design and procedure

Participants had to indicate whether the sentence with the quantifier: *most*, *many*, *few*, *fewer than half*, or *more than half* was true or false based on the sentence containing a proportion ranging from 1% to 99% (excluding 50%) (cf. Deschamps et al., 2015; Hackl, 2009; Pietroski et al., 2009; Schlotterbeck et al., 2020). We did not include the proportion 100%, because Ariel (2003) showed that *most* has an upper bound on meaning, and using it with 100% proportion is not accepted, although it is highly accepted with 99%. The upper bound of *most* could cause a divergence in the logistic function which we used in our model. We did not include 50%, because this proportion could be confusing for *more than half* and *fewer than half*.

While *most*, *more than half* and *fewer than half* have a proportional interpretation (Hackl, 2009), as explained above, *many* and *few* are ambiguous between cardinal reading (more/less than a certain number) and proportional reading (more/less than a certain proportion) (Partee, 1989). We used explicit partitive ‘of the’ and present proportions as a percentage for all quantifiers to ensure the proportional reading and avoid confusion for ambiguous quantifiers. Moreover, by using the percentage format we enforced the precise comparison between the proportion and the threshold. In this way, we minimized the differences between quantifiers in verification strategies. For example, in some experimental paradigms *most* is verified using an approximation strategy (Pietroski et al., 2009), while in others mixtures of strategies are used (Talmina et al., 2017).

The experiment started with a training block to familiarize participants with the procedure. In the training block, we used quantifiers *all, some, none* in the first sentence, which were not used in the actual experiment. Next, participants completed 250 trials (50 per quantifier) in randomized order. At the end of the experiment, participants provided basic demographic information. Each trial of the experiment consisted of two sentences displayed on separate screens. The first sentence containing the quantifier was of the form “[*Most/Many/Few/More than half/Fewer than half*] of the gleerbs are fizzda." To read this sentence participants had to press the arrow down key and keep it pressed. When they advanced to the next screen, they read a sentence containing proportion e.g., “20% of the gleerbs are fizzda." The proportion was drawn randomly, however, for quantifiers *more than half*, *fewer than half*, and *most* for which the 50% threshold was expected, we balanced proportions above and below 50% (25 proportions above and 25 proportions below 50%). Participants had to respond by pressing the right or left arrow keys corresponding to true or false judgments (counterbalanced between participants). In addition to participants’ judgments, we also collected response time data. Response times were measured from the onset of the second sentence until response.

In our experiment, we used pseudowords generated from 50 English six-letter nouns and adjectives using *Wuggy* software (Keuleers & Brysbaert, 2010). We used pseudowords to avoid pragmatic effects associated with quantifiers. The original words were controlled for frequency (*Zipf* value 4.06, van Heuven et al. (2014)). A native English speaker assessed the pseudowords in terms of how well they imitated English words.

### Computational model

The model was specified as a Bayesian hierarchical model. Let *i* indicate participants, *i* = 1, ..., *I*, *j* indicate the quantifier, *j* = 1, ..., 5, and *k* indicate the trial for each quantifier, *k* = 1, ..., $K_{ij}$. Then $Y_{ijk}$ is the *i*-th participant’s response to the *j*-th quantifier in the *k*-th trial, and $Y_{ijk} = 1$ if participant indicated true, and $Y_{ijk} = 0$ if participant indicated false. Then, we may model $Y_{ijk}$ as a Bernoulli, using the logit link function on the probabilities:

2 $$\begin{aligned} Y_{ijk} \sim \text {Bernoulli}(\pi _{ijk}) \end{aligned}$$

where the probability space of $\pi$ maps onto the $\mu$.

3 $$\begin{aligned} \pi _{ijk} = \gamma _{ij} + (1-2\gamma _{ij})\text {logit}^{-(\mu _{ijk}}) \end{aligned}$$

The additional parameter $\gamma _{ij}$ determines the probability of making a response error on either side of the threshold, namely erroneously saying true, or erroneously saying false. Each participant-quantifier combination has its own response error parameter estimate. The parameter $\mu _{ijk}$ has a linear model explication:

4 $$\begin{aligned} \mu _{ijk} = \frac{c_{ijk} - \beta _{ij}}{\alpha _{ij}} \end{aligned}$$

where $c_{ijk}$ indicates the percentage centered at 50%, parameters $\beta _{ij}$ indicate the threshold, and parameters $\alpha _{ij}$ correspond to the vagueness of the quantifier.

We defined prior probabilities on response error ($\gamma$), threshold ($\beta$), and vagueness ($\alpha$) parameters:

5a $$\begin{aligned} \gamma _{ij} \sim \text {Beta}(2, 20) \end{aligned}$$

5b $$\begin{aligned} \beta _{ij} \sim \text {Normal}(\delta _j, \sigma ^2_j) \end{aligned}$$

5c $$\begin{aligned} \alpha _{ij} \sim \text {log-normal}(\nu _j, \sigma ^2_{\alpha _j}) \end{aligned}$$

5d $$\begin{aligned} \nu _j \sim \text {Normal}(0, 5^2) \end{aligned}$$

5e $$\begin{aligned} \sigma ^2_{\alpha _j} \sim \text {Inverse-gamma}(2, 0.2) \end{aligned}$$

5f $$\begin{aligned} \sigma ^2_j \sim \text {Inverse-gamma}(2, 0.2) \end{aligned}$$

5g $$\begin{aligned} \delta _j \sim \text {Normal}(0, 5^2) \end{aligned}$$

The hierarchical nature of the distributions for $\alpha _{ij}$ and $\beta _{ij}$ indicate that we estimated the effect of threshold and vagueness for each participant under the assumption that they had a common mean and variance. The vagueness and threshold priors were fairly uninformative to avoid the inclusion of incidental constraints. Vagueness ($\alpha _{ij}$) came from a log-normal distribution to ensure only the positive estimates. Its mean ($\nu _j$) had a normal distribution, and its variance ($\sigma ^2_{\alpha _j}$) was drawn from the inverse-gamma distribution, as this distribution is typically used to model variance. For the thresholds ($\beta _{ij}$) we used a normal distribution with a common, normally-distributed mean ($\delta _j$) and the same variance distribution ($\sigma ^2_j$) as for $\alpha _{ij}$. The response error ($\gamma _{ij}$) came from a more informed distribution with most of its mass below an error rate of 20% for each true and false response^2^.

We used a hierarchical Bayesian model to estimate the parameters for each participant-quantifier combination. To fit the model, we used the *rstan* package in R (Stan Development Team, 2017) with six chains, 750 warm-up iterations per chain, and 2500 iterations per chain. Convergence for the model was not ideal, but after running 15000 iterations there were no divergent transitions and Rhats were within a reasonable range for all parameters (mostly < 1.05).

### Cluster analysis and correlations between parameters

Concerning between-subject consistency in vague quantifier-to-number mapping, we computed the difference in thresholds between pairs of vague quantifiers for each participant. Concerning how stretched the mental line of quantifiers is, we tested if the distance between thresholds of vague quantifiers was the same for all participants. This property is essential to establish what type of scale quantifiers create (e.g., rank vs. interval scale).

To investigate if subgroups of participants can explain the variability in thresholds, we ran an exploratory cluster analysis for the threshold parameter of all quantifiers^3^ estimating the clusters using agglomerative hierarchical clustering which groups observations into clusters based on their similarity. We chose this method because it does not require defining a specific number of clusters upfront. An additional advantage of hierarchical clustering is that it provides a hierarchical structure of the distance between observations which allows for more qualitative interpretation of the data. Because the number of participants entering the analysis was relatively small for the clustering method, we anticipated that some clusters might contain only a few participants. Therefore, we intended to use clustering to help us in the interpretation of individual differences rather than ultimately determining the number of subgroups. We also provided the interpretation of small clusters if the constellation of thresholds in these subgroups was meaningful in the light of linguistic theories. We used the Euclidean distance measure suitable for continuous input variables and the Ward linkage method ((Murtagh & Legendre, 2014), *hclust* function in R with *ward.D2* method) which minimizes variance inside the clusters.

**Table 1:** Mean (*SD*) parameters of individual participants for each quantifier, and additionally for threshold parameter the percent corresponding to mean thresholds

	Threshold	Vagueness	Response error
Few	–.103 (.073), 39.7%	.016 (.001)	.062 (.042)
Fewer than half	–.006 (.027), 49.4%	.002 (.00004)	.074 (.047)
Many	–.061 (.094) 43.9%	.019 (.003)	.048 (.024)
More than half	.001 (.012) 50.1%	.001 (.00003)	.042 (.019)
Most	.029 (.056) 52.9%	.009 (.001)	.047 (.024)

To assess the contribution of quantifier thresholds to the clustering, we performed a linear discriminant analysis (LDA). We used the stepwise procedure Wilks’ lambda assessment (*greedy.wilks* function in R package *klaR*, Roever et al. (2015)) to determine which variable contributed significantly to cluster formation. Next, we ran the LDA (*lda* function in R package *MASS*) to test how accurately the selected variables could predict the clusters. To validate the LDA, we ran leave-one-out cross-validation.

Finally, we tested whether there were any systematic patterns of correlations between parameters within quantifiers. Significant high correlations between the parameters of our model would mean that the parameters do not capture the unique source of variability in the data. Thus a more parsimonious model would be desired.

## Results

### Data pre-processing

We excluded 19 participants based on three exclusion criteria. Firstly, we excluded 11 participants who had 50% or more response times faster than 300 ms. Secondly, we excluded seven participants who failed to obey the monotonicity of quantifiers, defined in the following way: for positive quantifiers (*many*, *most*, and *more than half*) we expected the probability of providing the true response to increase with increasing proportion. The opposite effect should hold for negative quantifiers. To apply this criterion, we fitted the generalized linear model to participants’ response data with the proportion as a predictor and with by-subject random intercept and slope for proportion (*glmer* R function, Kuznetsova et al. (2017)). We excluded participants, who had a negative slope for positive quantifiers or a positive slope for negative quantifiers. Finally, we excluded one participant, who previously participated in a similar experiment. We excluded trials with response times shorter than 300 ms and longer than 2500 ms (similar cut-offs to Ratcliff and McKoon (2018)). This exclusion criterion aimed to exclude the fast guessing responses and the trials when participants drifted attention away from the task. Because we used pseudowords in our experiment, we anticipated that after a few trials, participants would only read carefully the quantifier (in the first sentence) and proportion (in the second sentence). Therefore, we expected that participants would perform the task fast. This justifies the 300-ms exclusion threshold. Moreover, 2.5 s was sufficient time to process the information about the proportion and execute the response. Altogether, we excluded 6% of trials, 1% of fast guessing and 5% of long responses. To be able to fit the same logit model to all quantifiers we flipped the true and false responses for *few* and *fewer than half*.

### Estimated parameters

**Fig. 2:**
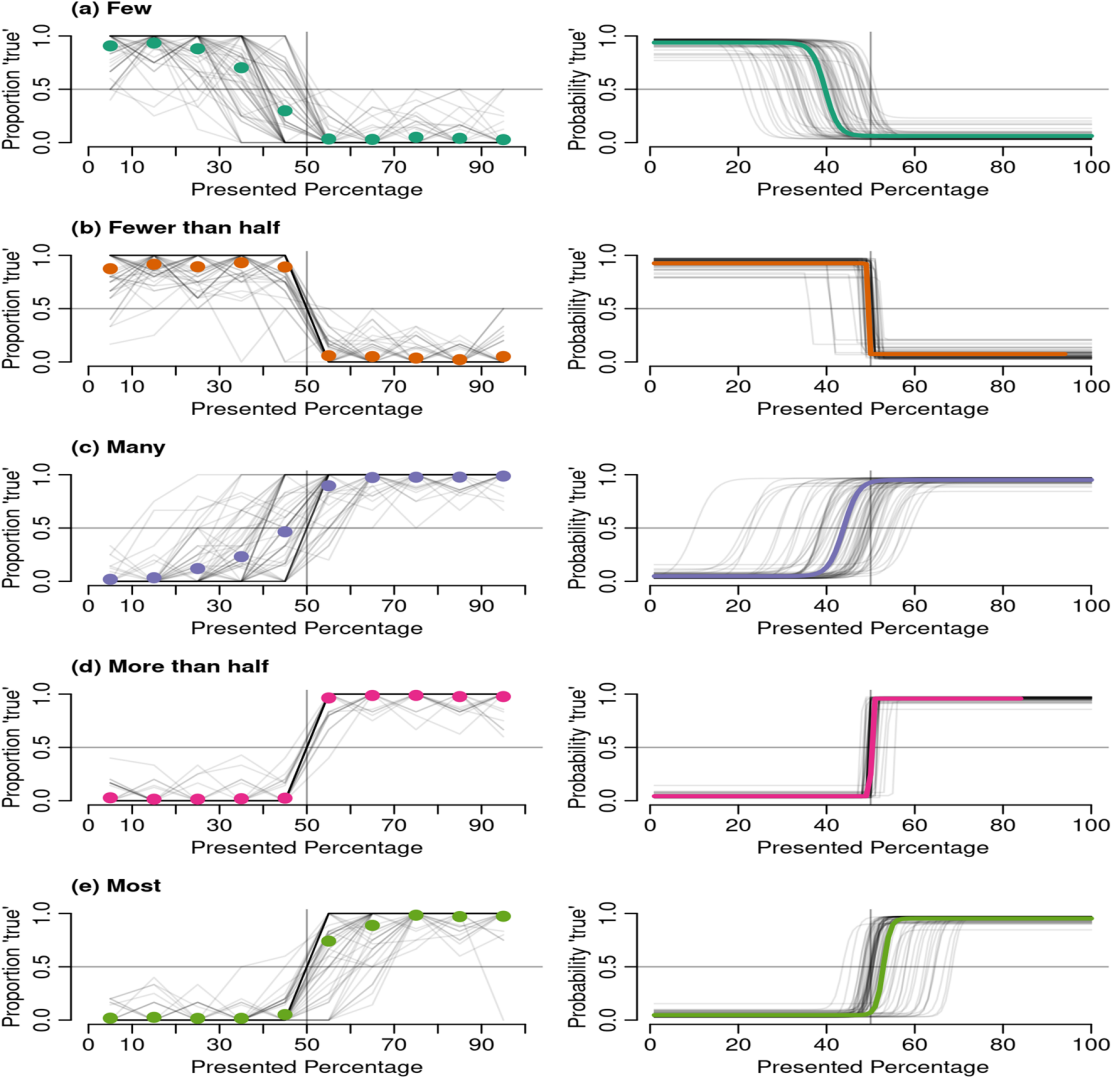
The *left panels* show the response data from participants. The *gray lines* represent individual participants and the *dots* represent aggregated binned responses (e.g., the first dot is the proportion of true responses for presented percentages between 0 and 10). The *right panels* show the logit curves estimated for each quantifier. The *colored lines *indicate the mean curves and the *gray lines* represent individual participants. The logit curves for the *few* and *fewer than half* were plotted consistently with the raw data

Table 1 shows the mean estimated model parameters. Figure 2 shows the estimated item response curves for each participant-quantifier combination; the overall response curves for the quantifiers are represented by the bold lines. We found greater individual variation in thresholds for *most*, *many*, and *few*, compared to *more than half* and *fewer than half*. At the group level, quantifier thresholds were represented in the following order (Friedman test $\chi ^2(4) = 134$, *p* $< 0.001$, moderate effect size *W* = 0.47): *few* had the lowest threshold, followed by *many*, then were *fewer than half* and *more than half*, and *most* had the highest threshold (pairwise comparison, Wilcoxon signed-rank test with Bonferroni correction).

**Fig. 3:**
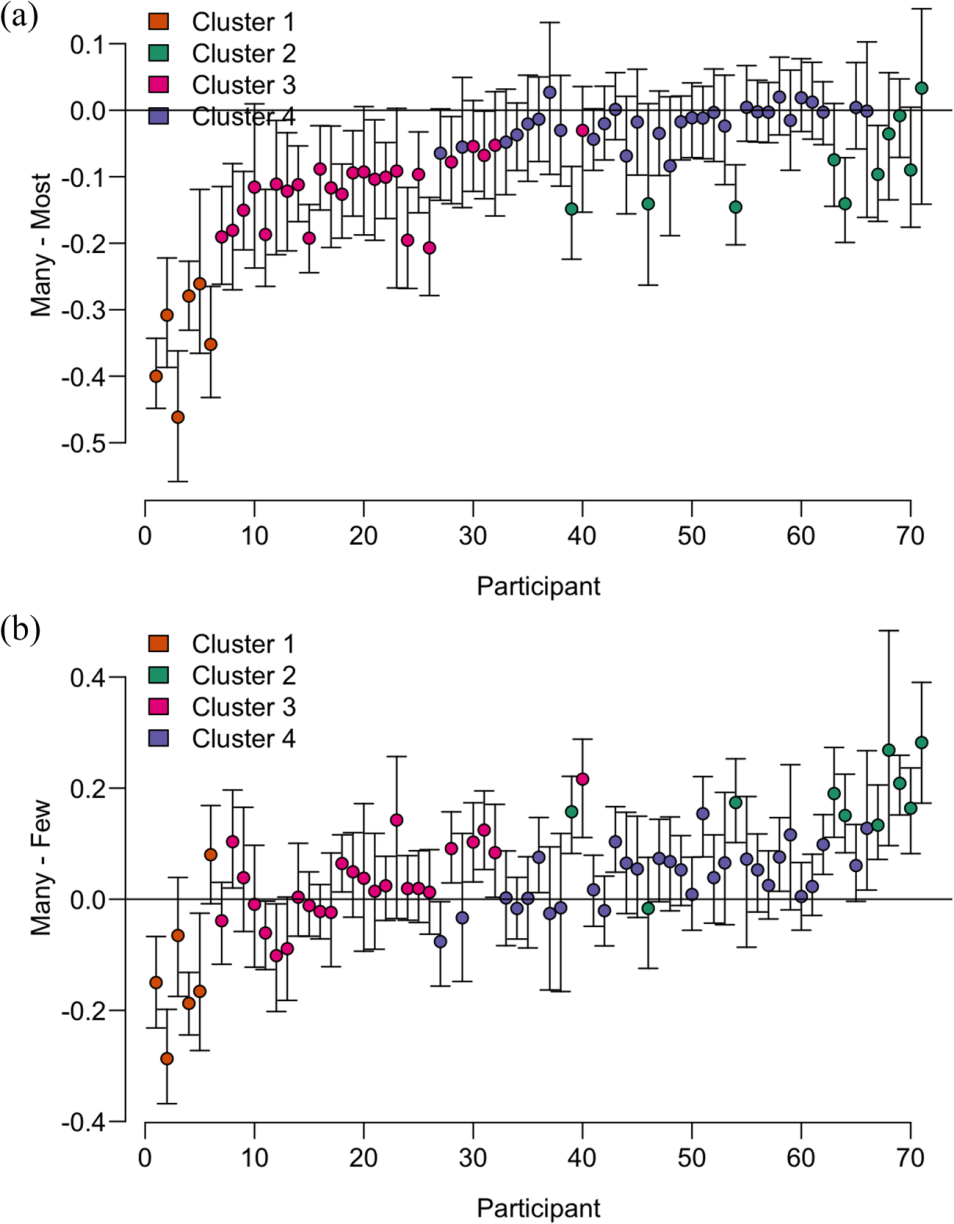
Differences between individual thresholds. The *error bars* indicate the 95% credible intervals. All participants are ordered by the posterior mean of their threshold for *many*. **(a)** The difference between the threshold for *many* and *most*. **(b)** The difference between the threshold for *many* and *few*. Colors are used to indicate cluster membership: Cluster 1 (*N* = 6) in *orange*, Cluster 2 (*N* = 10) in *green*, Cluster 3 (*N* = 25) in *pink*, and Cluster 4 (*N* = 30) in *purple*

The quantifiers *fewer than half* and *more than half* were the least vague as indicated by the steep response curves in Fig. 2. Moreover, *few* was more vague than *fewer than half* (*V* = 2556; *p* $< 0.001$), *many* was more vague than *more than half* (*V* = 2556; *p* $< 0.001$), *many* was more vague than *most* (*V* = 2556; *p* $< 0.001$), and *most* was more vague than *more than half* (*V* = 2556; *p* $< 0.001$). We also found that *fewer than half* had a greater response error than *more than half* (*V* = 2323; *p* $< 0.001$), and *few* had greater response error than *many* (*V* = 1809; *p* = 0.002). All *p* values based on Wilcoxon signed-rank test.

### Mental line of quantifiers

Concerning the order of vague quantifiers, Fig. 3a (the colors indicate cluster membership of each participant) shows that while all participants had lower or equal thresholds for *many* than for *most*, the distance between thresholds differed substantially between participants. Figure 3b, in turn, shows that many participants had similar thresholds for *many* and *few*, some participants had higher thresholds for *many* than for *few*, and some had lower thresholds *many* than for *few*.

### Cluster analysis results

We interpret the hierarchical clustering result as indicating four subgroups of participants with different constellations of thresholds (Fig. 1 in Supplementary materials). The four clusters were indistinguishable for the quantifiers *fewer than half* and *more than half* but differed substantially in thresholds for the quantifiers *few*, *many*, and *most* (see Table 2 and Fig. 3).

**Table 2:** Mean (*SD*) threshold parameter in each cluster and percentage corresponding to mean thresholds, four-cluster solution

Quantifier	Cluster 1	Cluster 2	Cluster 3	Cluster 4
	(*N* = 6)	(*N* = 10)	(*N* = 25)	(*N* = 30)
Few	–.16 (.11)	–.13 (.07)	–.14 (.06)	–.05 (.04)
	34.5%	36.5%	36.2%	44.6%
Fewer than half	–.01 (.01)	–.0001 (.01)	–.02 (.02)	–.01 (.03)
	49.9%	50.0%	49.8%	48.7%
Many	–.28 (.01)	.03 (.01)	–.11 (.04)	–.012 (.03)
	21.5%	53.7%	39.3%	48.8%
More than half	–.004 (.01)	–.002 (.01)	.001 (.01)	.002 (.01)
	49.6%	49.8%	50.1%	50.2%
Most	.06 (.08)	.12 (.04)	.012 (.04)	.006 (.03)
	55.9%	62.1%	51.1%	50.5%

Participants in Cluster 4 (*N* = 30) had the lowest threshold for *most*, and the highest for *few*, while participants in Cluster 2 (*N* = 10) had the highest threshold for *most* and *many*. Participants in Cluster 1 (*N* = 6) had lower threshold for *many* than for *few*.

We found that only vague quantifiers contributed to the clustering: *many* ($\lambda$ = 0.15, *p* < 0.001), *most* ($\lambda$ = 0.07, *p* < 0.001), and *few* ($\lambda$ = 0.06, *p* < 0.001). The LDA accuracy in classification into Clusters 1 to 4 based on thresholds for *many*, *few* and *most* was 100%, and the leave-one-out cross-validation accuracy was 96%.

### Correlations between vagueness, threshold, and response error

We tested the correlations between vagueness, threshold, and response error parameters of the model (Supplementary materials, Fig. 4). We found significant correlations between threshold and vagueness for *few* (*r* = -0.33), *many* (*r* = -0.31), and *more than half* (*r* = 0.30), between threshold and response error for *fewer than half* (*r* = -0.32), and response error and vagueness for *many* (*r* = 0.53) and *most* (*r* = 0.52).

## Discussion

In this study, we investigated between-subjects variability in quantifier-to-number mapping by means of a computational model. We found that vague quantifiers had a higher vagueness value and that negative quantifiers had a higher response error value. Moreover, we found individual differences in thresholds for *many*, *few*, and *most*. A cluster analysis explains the differences between participants by grouping them into four clusters. In all groups, *most* had the highest threshold, which is compatible with the analysis of *most* as a superlative of *many* (*many-est*, Hackl (2009)). However, the mean threshold of *most* varied between clusters (50.5% in Cluster 4 and 62% in Cluster 2). The members of Cluster 4 kept the threshold for *many* close to *most*, while members of Clusters 1, 2, and 3 kept a larger distance between the thresholds (see Fig. 3a). Moreover, the vast majority of participants judged *few* as less than *many*. However, they disagreed on the numerical distance between the thresholds of these quantifiers. This finding indicates the quantifier scale is of rank type.

The cluster analysis revealed that subgroups differed in the semantics of vague, polar-opposite quantifiers (*few* and *many*). The mean thresholds in Clusters 3 and 4 are compatible with the interpretation of *few* as a negation of *many*. While the mean thresholds of *few* and *many* were higher in Cluster 4 than in Cluster 3, the numerical distance between the thresholds of polar-opposite quantifiers was small in both clusters.

In contrast, the semantics of vague, polar-opposite quantifiers in Clusters 1 and 2 lead to two forms of borderline contradiction (Égré & Zehr, 2018, cf. Ripley, 2009). Borderline contradictions arise when a vague predicate P and its negation are asserted or denied about the same entity (conjunctive case "x is P and not P" or disjunctive case "x is neither P nor not P", cf. Égré & Zehr, 2018). Our analysis shows that this phenomenon extends to vague, polar-opposite quantifiers. The conjunctive type of borderline contradiction leads to glutty semantics (Égré & Zehr, 2018), as in Cluster 1, whose members accepted certain proportions as *many and few*. The disjunctive type leads to gappy semantics (Égré & Zehr, 2018), as in Cluster 2, whose members accept certain proportions as *neither many nor few*. The glutty semantics of vague quantifiers endorsed by participants in Cluster 1 go against the prediction of between-subject consistency in the order of quantifiers.

The greater flexibility of *many* on the mental line as compared to *few* cannot be explained by its context-dependency. First, in our experiment, we used an artificial context by introducing pseudowords. There was no reason for participants to have different expectations about the context. Second, the low-magnitude quantifiers are more context-dependent than high-magnitude quantifiers, for example, they can have different thresholds depending on the reference set (Newstead et al., 1987). This means that different expectations about the context would lead to greater variation in thresholds for *few* than *many*.

We attribute this asymmetry in threshold flexibility to semantic competition between quantifiers. At the lower bound *many* competes with *few*, while at the upper bound with *most*. These two constraints resulted in a different stretch of quantifier mental line between subgroups. Participants in Cluster 4 had the most shrunk mental line, ranging between 44% and 50%. In contrast, participants in Clusters 2 and 1 had stretched mental lines, ranging between 36% (21%) and 62% (55%). The mental line in Cluster 3 stretched moderately between 36% and 51%.

Concerning the relationship between the three model parameters, the only significant correlation between threshold and response error was for *fewer than half* (however, strongly affected by the outlier participants, see Supplementary materials Fig. 5). The lack of correlations between response error and threshold shows that the variation in thresholds reflects variation in the quantifier-to-number mapping and it is not an artifact of task performance. Although the correlation between vagueness and response error was more consistent (at least in direction) than between the other parameters, the lack of systematic pattern (only significant for *many* and *most*) shows that they correspond to two different processes that should be modeled by separate parameters. Vagueness thus may correlate with response error for some quantifiers, but it cannot be equated with threshold-independent erroneous responding (cf. Denić and Szymanik, 2022). Relatedly, the overall magnitude of the vagueness parameters was quite small. One reason for both the correlations and the low magnitude might be an issue in identifiability in the model.

To summarize, computational modeling has been proven useful in testing the meaning representations of quantifiers predicted by different semantic theories. For example, van Tiel et al. (2021) showed that bivalent truth-conditional semantics can account for the meaning of quantifiers equally well as prototype semantics when supplied with the pragmatic interpretation of meaning. Our modeling uncovered an additional challenge to the truth-conditional semantics approach (Barwise & Cooper, 1981), individual differences in thresholds. Égré (2017) argued that speakers can faultlessly disagree when evaluating sentences involving vague adjectives (e.g., John is *tall*.) for three reasons. They may disagree about the comparison class (e.g., children vs. basketball players), standard (which height is representative for a given comparison class), or criteria. While context can resolve the disagreement in the first two cases, the last case directly relates to the semantic representation of threshold. Our investigation shows that even in an abstract context, participants substantially differ in quantifier-to-number mapping. Moreover, in the task in which there was little uncertainty about the numerical information, the individual differences in imprecise number representation played a minor role. While we found that vague quantifiers had a higher value of the vagueness parameter than other quantifiers, between-participants variability in the vagueness was small (except for *many*, see Supplementary materials).

Bivalent truth-conditional semantics (Barwise & Cooper, 1981) are difficult to reconcile with individual differences in thresholds and with the demonstrated vagueness of some quantifiers. In contrast, fuzzy logic (Zadeh, 1983) predicts vagueness and can incorporate individual differences by allowing for graded truth values. In this view, the meaning of a quantifier could be a function of the averaged truth value judgments of this quantifier for each proportion. However, this approach obscures the distinction of two sources of between-participant variability: vagueness and threshold. In our study, we found significant correlations between these parameters for *more than half*, *many*, and *few*, but not for *most* and *fewer than half*. Therefore, our finding supports the conceptual distinction between these sources, where vagueness in criteria corresponds to threshold parameter and vagueness in degree to vagueness parameter (cf. Devos, 1995).

In this paper, we presented a novel approach to study truth-conditional meanings of quantifiers, while controlling for factors such as response error and vagueness. We validated our approach by showing quantitative individual differences in truth conditions of vague quantifiers, as exemplified by different quantifier-to-number mapping, and qualitative differences in the semantics of vague quantifiers, as exemplified by gappy vs. glutty semantics of polar-opposite quantifiers. Further extension of our modeling approach can make a two-fold contribution. On the linguistic side, the model can be used to test individual differences in many other natural language categories, including gradable adjectives (Verheyen & Égré, 2018; Verheyen et al., 2018), semantic categorizations of nouns Verheyen and Storms (2013); Verheyen et al. (2018, 2019), probability terms (Wallsten et al., 1986; Mosteller & Youtz, 1990; Schuster & Degen, 2019), and presuppositions projection (Sudo et al., 2012). On the psychology side, the model can be applied to test the structure of other quantifier scales often used in psychometrics such as frequency or probability terms scales. In sum, in this paper, we showed that our computational model can bring together formal semantic and psycholinguistic approaches to study meaning representations.

## Open Practices Statement:

The data and analysis code are available at https://github.com/jstbcs/pling-quant. The experiment reported in this paper was not preregistered.

## Supplementary Information

Below is the link to the electronic supplementary material.

Supplementary file 1 (pdf 1153 KB)

## Data Availability

The data are available at https://github.com/jstbcs/pling-quant.

## Footnotes

1 In this paper, we focus only on quantifiers with one threshold. Some quantifiers can have two or more thresholds, e.g., *between 3 and 6* has two thresholds, 3 and 6.

2 To reduce the complexity of the model, we did not use hierarchical modeling for response errors.

3 See cluster analysis for vagueness and response errors in Appendix.

## Code availability

The analysis code are available at https://github.com/jstbcs/pling-quant.
