# Supplementary material for: Most quantifiers have many meanings
Source: Psychon Bull Rev. 2024 May 8;31(6):2692–703. doi: 10.3758/s13423-024-02502-7 (PMC11680628; doi:10.3758/s13423-024-02502-7)
Supplement: Supplementary file 1 — (pdf 1153 KB) [file 13423_2024_2502_MOESM1_ESM.pdf]

Supplementary materials for "*Most* quantifiers have *many* meanings"

Sonia Ramotowska[a], Julia Haaf[b], Leendert Van Maanen[c], and Jakub Szymanik[d]

[a]Institute for Logic, Language and Computation, University of Amsterdam, Science Park 107, 1098 XG Amsterdam, the Netherlands
[b]Department of Psychology, Karl-Liebknecht-Str. 24/25 14476 Potsdam, Germany
[c]Department of Experimental Psychology & Helmholtz Institute, Utrecht University, Heidelberglaan 1, 3584 CS Utrecht, The Netherlands
[d]Center for Mind/Brain Sciences Department of Computer Science, University of Trento, Corso Bettini 31, 38068 Rovereto (TN), Italy

For Review Only

1 Supplementary materials

1.1 Threshold

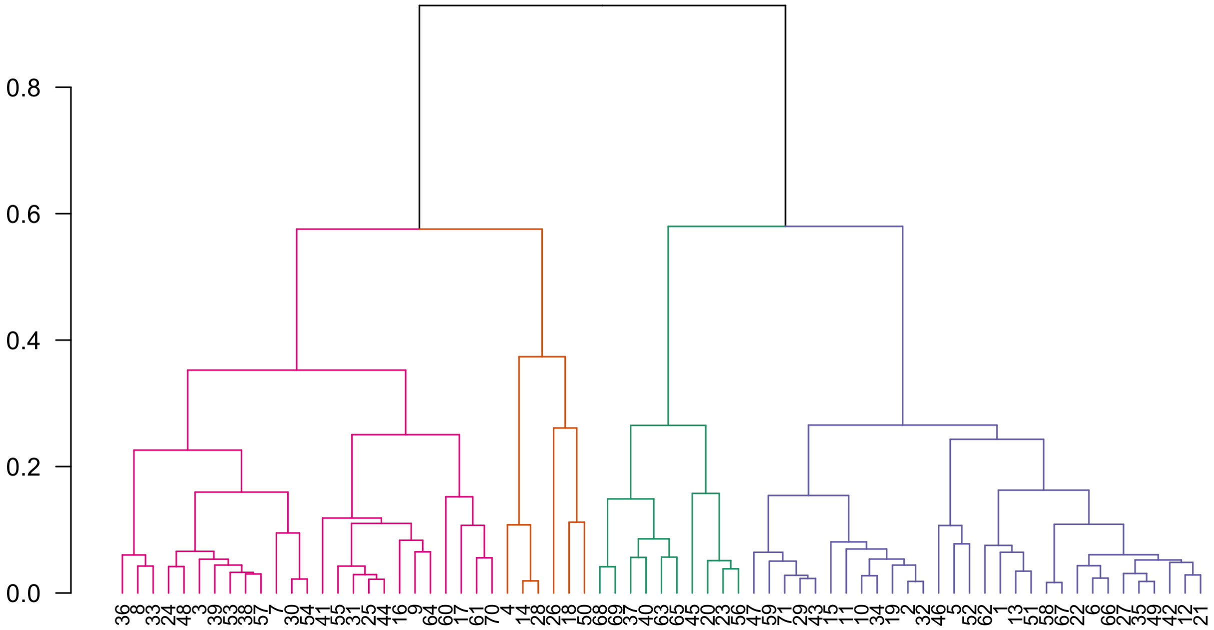

Figure 1: Dendrogram showing four clusters based on threshold parameters. Colors are used to indicate the cluster membership (by cluster size): Cluster 1 ($N = 6$) in orange, Cluster 2 ($N = 10$) in green, Cluster 3 ($N = 25$) in pink, and Cluster 4 ($N = 30$) in purple. The y-axis shows the height of the dendrogram and the x-axis the IDs of participants in each cluster.

1.2 Vagueness

1.2.1 Cluster analysis

We expected that participants could differ in the perceived vagueness of vague quantifiers. Hierarchical clustering analysis helped us identifying one cluster ($N = 15$) with high vagueness for *many*, and a second cluster ($N = 56$) with lower vagueness for *many*. We report here the simpler 2-cluster solution (see Table 1), however, we noted that the larger cluster consisted of two sub-clusters (see Figure 2).

Table 1: Mean (SD) vagueness parameter in each cluster, 2-cluster solution.

Quantifier	Cluster 1 ($N = 15$)	Cluster 2 ($N = 56$)
Few	.016 (.001)	.016 (.001)
Fewer than half	.002 (.00003)	.002 (.00004)
Many	.024 (.002)	.018 (.002)
More than half	.001 (.00004)	.001 (.00002)
Most	.009 (.001)	.009 (.001)

1.2.2 Linear Discriminant Analysis

For the vagueness parameter, we expected vague quantifiers to contribute to the clustering. We found that *many* ($\lambda = 0.30$, $p < 0.001$) and *most* ($\lambda = 0.28$, $p = 0.03$) contributed significantly to the clustering. The LDA achieved 100% accuracy in the classification of participants into clusters based on vagueness parameters for *many* and *most*, and the leave-one-out cross-validation accuracy was 100%.

For completeness, we tested if *few* would contribute to clustering given the 3-cluster solution. We found that only the contribution of *many* and *most* was significant ($\lambda = 0.11$, $p < 0.001$; $\lambda = 0.09$, $p = 0.01$ respectively).

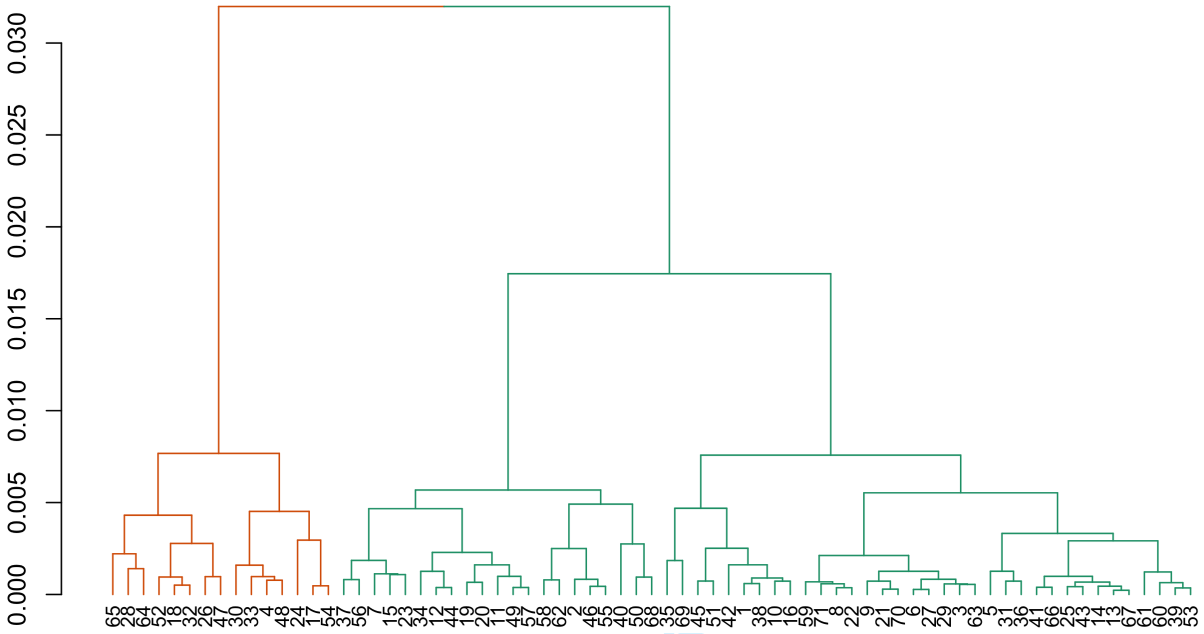

Figure 2: Dendrogram showing two clusters based on vagueness parameters. Colors are used to indicate the cluster membership: Cluster 1 ($N = 15$) in orange and Cluster 2 ($N = 56$) in green. The y-axis shows the height of the dendrogram and the x-axis the IDs of participants in each cluster.

1.3 Response error

1.3.1 Cluster analysis

The hierarchical clustering method separated participants into two clusters with few response errors ($N = 66$) and a cluster with more response errors ($N = 5$) across quantifiers, see Table 2 and Figure 3. This means that the majority of participants had a low response error rate. The difference in response error between clusters was most prominent for negative quantifiers.

Table 2: Mean (SD) response error parameter in each cluster, 2-cluster solution.

Quantifier	Cluster 1 ($N = 5$)	Cluster 2 ($N = 66$)
Few	.19 (.03)	.05 (.02)
Fewer than half	.20 (.02)	.06 (.03)
Many	.08 (.05)	.05 (.02)
More than half	.06 (.02)	.04 (.02)
Most	.10 (.03)	.04 (.02)

1.3.2 Linear Discriminant Analysis

We expected the response error parameter for negative quantifiers to contribute more to clustering. In line with this hypothesis, the Wilks test showed a significant contribution of response error parameters for *few* ($\lambda = 0.28$, $p < 0.001$) and *fewer than half* ($\lambda = 0.26$, $p < 0.001$), but not for *many*, *most* and *more than half*. We used the LDA to predict the cluster membership for each participant based on response error parameters for *few* and *fewer than half*. The LDA achieved 100% accuracy, and the leave-one-out cross-validation accuracy was 100%.

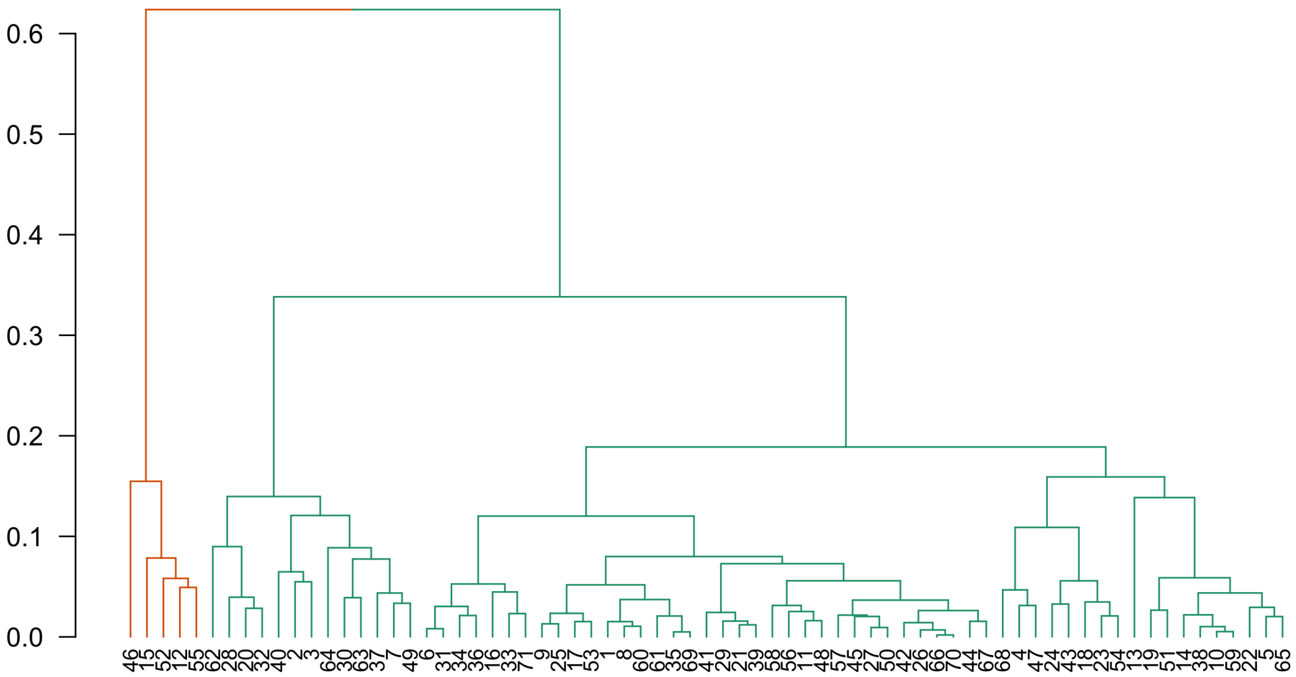

Figure 3: Dendrogram showing two clusters based on response error parameters. Colors are used to indicate the cluster membership: Cluster 1 ($N = 5$) with a high response error is in indicated in orange, and Cluster 2 ($N = 66$) with a low response error in green. The y-axis shows the height of the dendrogram and the x-axis the IDs of participants in each cluster.

Only

1.4 The correlations between vagueness, threshold, and response error

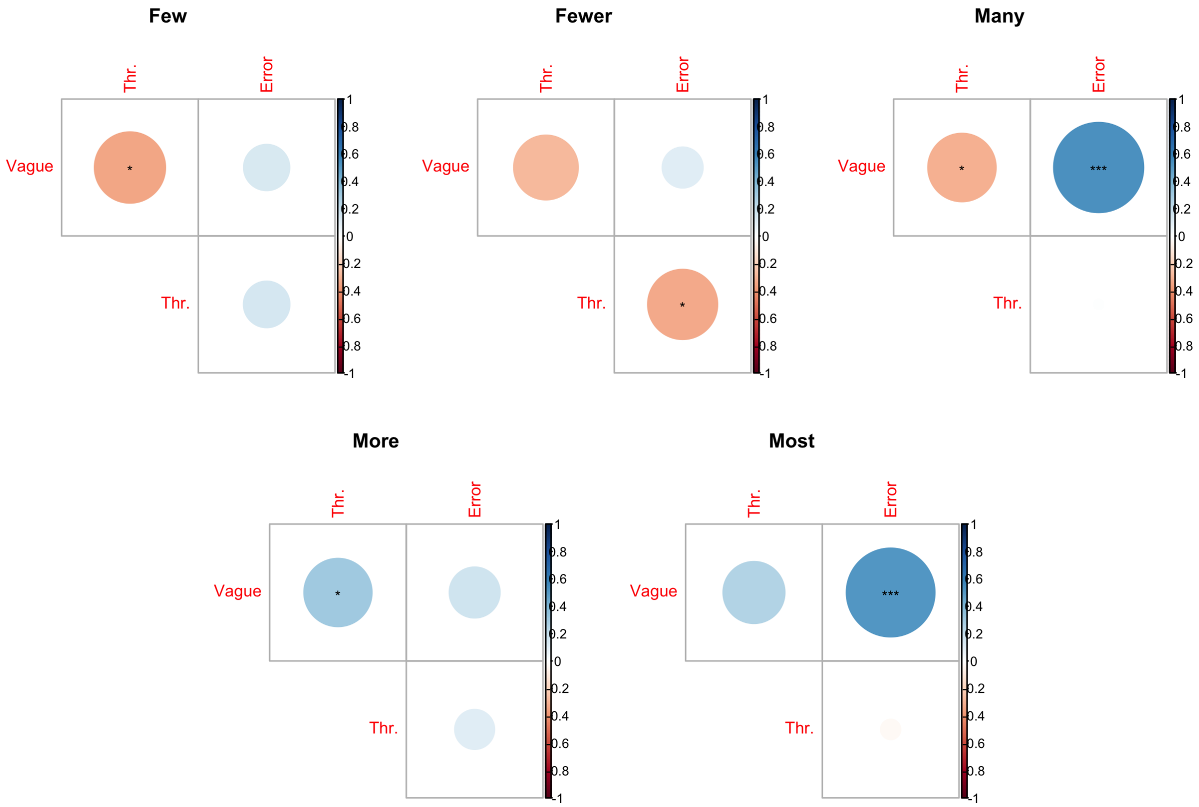

Figure 4: Correlations of parameters for each quantifier (significance level *** 0.001, ** 0.01, * 0.05). The p - values were adjusted using the Bonferroni correction.

1.5 Influential observations

Figure 11 illustrates how relationships between model parameters for each quantifier are affected by influential observations. We computed the Cook's distance using the *ols plot cooksd bar* R function in the package *olsrr* (Hebbali, 2020).

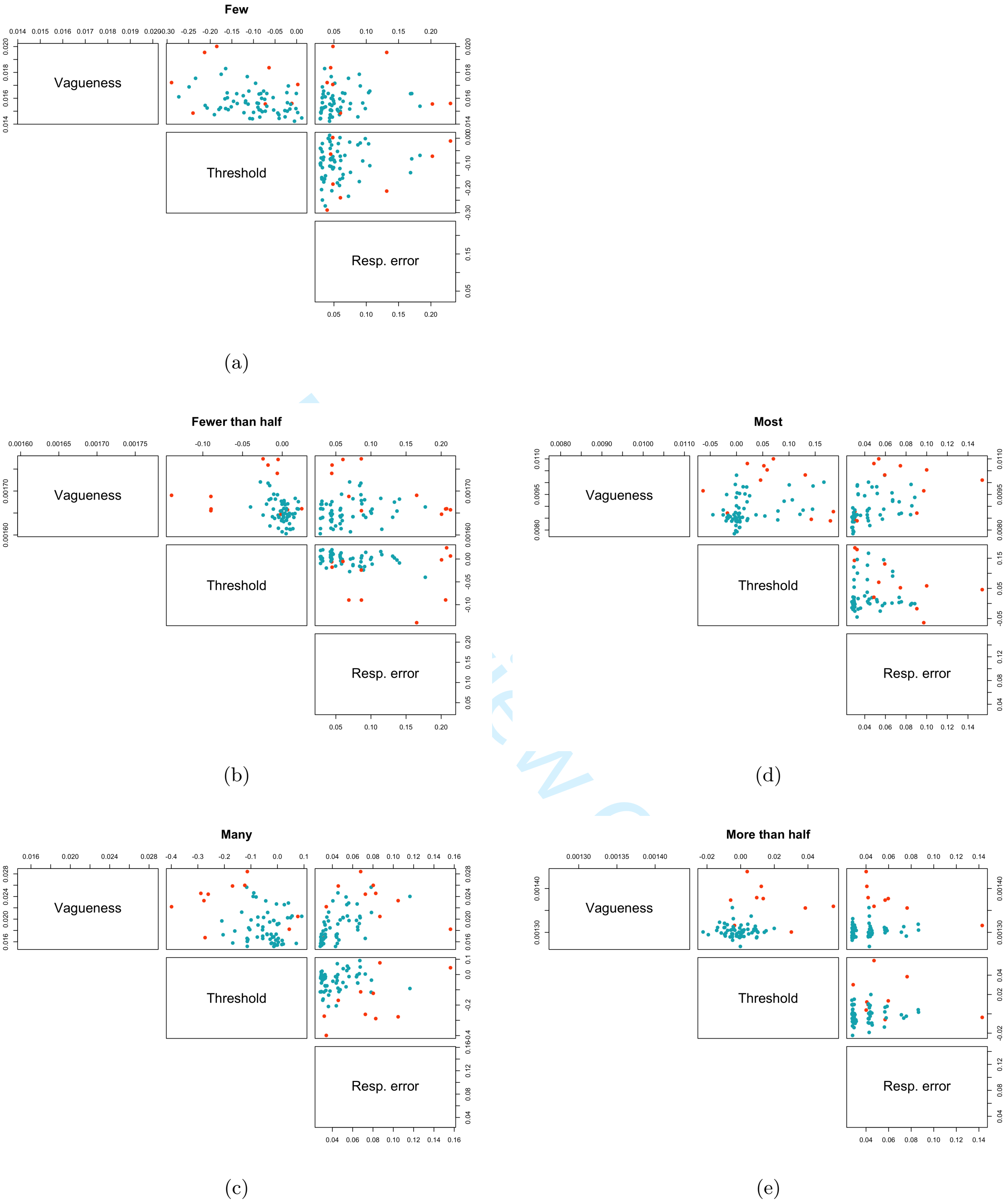

Figure 5: The scatter plots illustrate the relationships between model parameters (abbreviation Resp. error - response error) for each quantifier. The influential observations according to Cook's distance are indicated in red.

References

Hebbali, A. (2020). Tools for Building OLS Regression Models [Computer software manual]. (R package

version 0.5.3)

For Review Only
